# Intratumoral heterogeneity derived from synthetic MRI for characterization of HER2 subtypes in breast cancer

**DOI:** 10.3389/fonc.2026.1801894

**Published:** 2026-08-28

**Authors:** Junjie Zhang, Ligang Hao, Luqi He, Xiaochong Zhang, Leting Su, Fengxiao Gao

**Affiliations:** 1 Department of Computed Tomography and Magnetic Resonance, Xingtai People’s Hospital, Xingtai, Hebei, China; 2 Department of Thoracic Surgery, Xingtai People’s Hospital, Xingtai, Hebei, China; 3 Department of Science and Education, Xingtai People’s Hospital, Xingtai, Hebei, China

**Keywords:** breast cancer, HER2, intratumoral heterogeneity (ITH), machine learning, magnetic resonance imaging

## Abstract

**Background:**

The human epidermal growth factor receptor 2 (HER2) plays a pivotal role in determining both the prognosis and therapeutic strategies for breast cancer patients. This study aimed to develop a habitat model based on synthetic magnetic resonance imaging (MRI) to quantitatively assess intratumoral heterogeneity (ITH) and to evaluate its value in predicting HER2 expression status.

**Methods:**

A retrospective analysis was conducted from September 2023 to September 2025, including 233 patients with pathologically confirmed invasive breast cancer. The investigation was organized into three key tasks: distinguishing HER2-positive from HER2-negative tumors; differentiating HER2-low from HER2-zero/positive cases; and separating HER2-zero from HER2-low/positive cases. Tumor regions were identified on synthetic MRI images and underwent clustering analysis. The resulting clusters, together with global pixel distribution patterns, were used to calculate ITH scores—specifically, ITHT1, ITHT2, and ITHPD. Patients were randomly allocated to either a training or validation set in a 7:3 ratio. Clinical independent predictors were identified through multivariable logistic regression and subsequently integrated with ITH signatures to construct comprehensive predictive models. Nine distinct machine learning algorithms were evaluated to determine the most effective approach. Model performance was assessed using the area under the receiver operating characteristic curve (AUC) as well as decision curve analysis (DCA). Additional evaluation metrics included the Kolmogorov-Smirnov (KS) statistic and calibration curves to appraise clinical utility.

**Results:**

The integrated model, developed using the random forest algorithm, demonstrated superior predictive capability across all three tasks. In Task 1, the model achieved AUC values of 0.855 and 0.835 for the training and validation sets, respectively. For Task 2, the model yielded AUCs of 0.905 and 0.801, while in Task 3, AUCs of 0.825 and 0.814 were observed in the training and validation sets, respectively. Notably, the model’s clinical utility was substantiated by favorable Brier scores and validation through KS statistics and DCA.

**Conclusions:**

The integration of clinical predictors with synthetic MRI-derived ITH signatures resulted in robust, noninvasive models for preoperatively predicting HER2 expression status in breast cancer patients.

## 1 Introduction

Breast cancer remains the predominant cause of mortality and the most frequently diagnosed malignancy among women globally. In 2022, approximately 2.3 million new cases of breast cancer were reported worldwide, resulting in an estimated 670,000 cancer-related fatalities (1). Approximately 15% to 20% of breast cancer patients exhibit HER2-positive expression, and the advent of HER2-targeted therapies has markedly improved patient survival rates (2). With the progression of antibody-drug conjugates, HER2-low expression has been proposed as a novel subtype, offering potential benefits through innovative targeted treatments. In 2022, Modi and colleagues reported that a novel antibody-drug conjugate targeting HER2 has the potential to improve progression-free and overall survival in patients with HER2-low breast cancer (3). This discovery has the potential to transform the conventional understanding of HER2 status. Nevertheless, it remains imperative to accurately evaluate HER2 subtypes in breast cancer.

Presently, HER2 expression is determined using immunohistochemistry (IHC) and fluorescence *in situ* hybridization (FISH) analyses of tissue samples (4). However, it is essential to acknowledge certain limitations, such as potential sampling bias and the invasive nature of these sampling techniques. Numerous prior studies have explored the prediction of HER2 expression in breast cancer utilizing MRI radiomics and deep learning (DL), time-dependent diffusion MRI, or synthetic MRI with quantitative parameters (5–9). These findings support the potential of employing non-invasive imaging techniques to distinguish HER2 status. While these methods have demonstrated efficacy in accurately assessing HER2 status, they frequently treat the tumor as a homogeneous entity, thereby overlooking its internal heterogeneity. Intratumoral heterogeneity (ITH) denotes the existence of distinct subpopulations of tumor cells within a primary tumor or between a primary tumor and its metastases, characterized by genetic, phenotypic, or behavioral variations (10). In the context of breast cancer, HER2 ITH is a well-documented phenomenon, observed in up to 40% of tumors. Notably, HER2 ITH is more prevalent in HER2-low breast cancers compared to HER2-zero or HER2-positive counterparts (11). Currently, the assessment of ITH predominantly relies on tissue sampling, pathological examination, and genomic sequencing. These methodologies, however, are invasive, prone to sampling bias, and inadequate for real-time ITH analysis. Therefore, there is a pressing demand for noninvasive and efficient approaches to evaluate ITH.

MRI is a highly effective non-invasive technique for the diagnosis, staging, and monitoring of various forms of breast cancer. MRI provides a comprehensive view of tumor tissue by capturing images that encompass both morphological and textural characteristics (12). ITH in breast cancer can manifest on MRI scans as either local or global variations. Radiomic habitat analysis serves as a non-invasive method for detecting this ITH by precisely segmenting tumors into subregions that reflect biological diversity (13). This technique has also been successfully employed to predict invasiveness in lung adenocarcinoma and to assess complete pathological response to neoadjuvant chemotherapy in breast cancer (14, 15). To date, no research has combined clinical independent predictors with an ITH score derived from synthetic MRI models to ascertain HER2 status in breast cancer. This study aims to investigate whether synthetic MRI (SyMRI)-based ITH score can effectively distinguish between HER2-zero, HER2-low, and HER2-positive tumors.

## 2 Methods

### 2.1 Study design and patient selection

The study was conducted in accordance with the Declaration of Helsinki and received approval from the Ethics Committee of Xingtai People’s Hospital (Ethics Committee reference number: 2026 (063), dated March 27, 2026). As the study was retrospective in nature, the requirement for written informed consent from the patients was waived.

The reporting of this study adheres to the TRIPOD guidelines. The research involved a consecutive review and retrospective analysis of all patient data with pathologically confirmed invasive breast cancer from September 2023 to September 2025 at Xingtai People’s Hospital. The inclusion criteria were as follows: (1) invasive breast cancer confirmed by surgical pathology; (2) acquisition of breast MRI, including SyMRI scans, within two weeks prior to the pathological diagnosis; and (3) HER2 status determined by IHC, with IHC 2+ status further evaluated by FISH analysis. The exclusion criteria included: (1) inadequate MRI acquisition due to motion artifacts; (2) receipt of antitumor therapy prior to MRI examination and pathological diagnosis; (3) previous or concurrent presence of other malignant tumors; and (4) incomplete clinical and histopathological data. For patients presenting with multiple lesions, the lesion with the largest diameter was selected for inclusion in the study.

The study ultimately included a total of 233 patients and was organized around three distinct tasks. Task 1 involved differentiating between HER2-positive and HER2-negative breast cancer. Task 2 focused on distinguishing HER2-low from HER2-zero/positive breast cancer, while Task 3 aimed to differentiate HER2-zero from HER2-low/positive breast cancer.

In Task 1, the cohort of 233 patients, comprising 83 HER2-positive and 150 HER2-negative individuals, was randomly divided into a training set (n = 163) and a validation set (n = 70), maintaining a 7:3 ratio. Similarly, in Task 2, the same cohort, consisting of 72 HER2-low and 161 HER2-zero/positive patients, was divided into a training set (n = 163) and a validation set (n = 70) following the same 7:3 ratio. In Task 3, the cohort, which included 78 HER2-zero and 155 HER2-low/positive patients, was again randomly divided into a training set (n = 163) and a validation set (n = 70), adhering to the 7:3 ratio.

### 2.2 Histopathological analysis

All histopathological assessments were independently evaluated by two pathologists, with any discrepancies adjudicated by the more experienced pathologist. The categorization of HER2 status was as follows: (1) HER2-positive, defined by an IHC score of 3+ or 2+ with a positive FISH result; (2) HER2-negative, characterized by an IHC score of 0, 1+, or 2+ with a negative FISH result; (3) HER2-low, indicated by an IHC score of 1+ or 2+ with a negative FISH result; and (4) HER2-zero, identified by an IHC score of 0. Tumors were classified as hormone receptor-negative if less than 1% of cells exhibited nuclear staining, while those with 1% or more were classified as hormone receptor-positive. The threshold for Ki-67 was established at 20%.

### 2.3 MRI image acquisition

In this study, a GE 3.0T MRI system (SIGNA Architect, GE Healthcare, Milwaukee, WI) was utilized in conjunction with an 8-channel receiver coil specifically designed for bilateral breast imaging. Patients were positioned prone, allowing their bilateral breasts to be naturally positioned within the coil to include the axillary regions. The standard MRI protocol included axial T1-weighted sequences, T2-weighted sequences with fat suppression, diffusion-weighted imaging (DWI) sequences (b = 0, 800 s/mm²), and dynamic contrast-enhanced MRI (DCE-MRI). Beyond the standard MRI protocol, all patients underwent axial synthetic MRI sequences following the administration of a contrast agent. The MAGnetic resonance image Compilation (MAGiC) technique was employed for synthetic MRI image processing on the GE 3.0 Tesla system, enabling the automatic computation of quantitative maps for T1, T2, and proton density (PD).

### 2.4 Image analysis

Two radiologists, possessing 5 and 14 years of experience in breast imaging respectively, independently reviewed all images without access to clinical or pathological information. Quantitative maps of T1, T2, and PD were generated using synthetic MRI sequences processed through the MAGiC post-processing software. Primary tumors were identified utilizing conventional MRI image data, and the largest diameter of each detected tumor was measured on DCE-MRI. In cases where multiple lesions were present, the largest was selected for assessment. The region of interest (ROI) was manually delineated in the solid area on the largest slice of each lesion on synthetic T2-weighted images, avoiding visible blood vessels, hemorrhage, necrosis, or cystic tumor regions. T1 relaxation time (T1), T2 relaxation time (T2) and PD values were automatically calculated for every voxel within the ROI for each subject, with the analysis based on the average values obtained from the two radiologists. Similarly, we set the ROIs and measured the apparent diffusion coefficient (ADC) values on the ADC maps in the same way.

### 2.5 ITH score calculation

**Figure 1:**
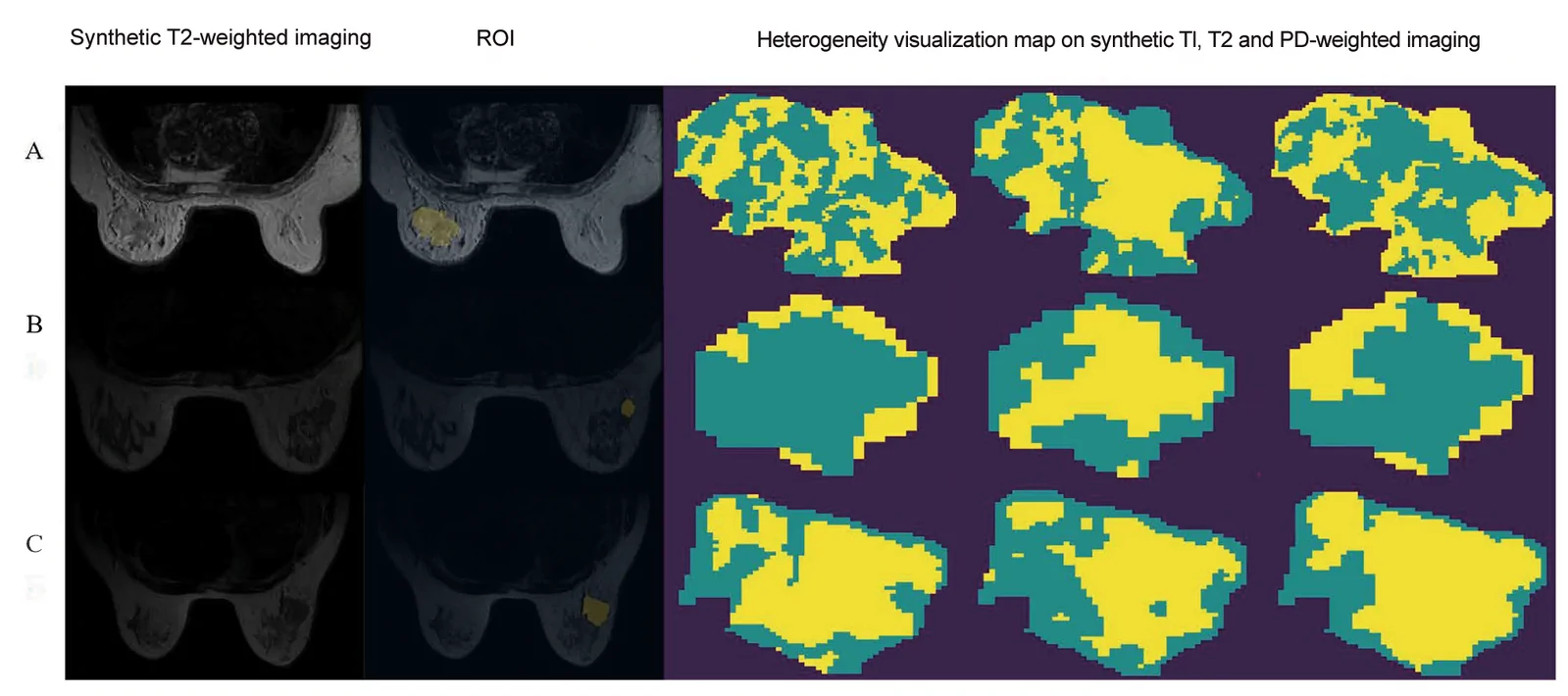
Illustrates representative synthetic T2-weighted images, ROI delineation, and intratumoral heterogeneity visualization in patients with HER2-zero, HER2-low, and HER2-positive breast cancers. **(A)** depicts a 68-year-old female patient diagnosed with HER2-positive breast cancer, with ITHT1, ITHT2, and ITHPD scores of 0.95, 0.92, and 0.90, respectively. **(B)** presents a 62-year-old female patient with HER2-low breast cancer, exhibiting ITHT1, ITHT2, and ITHPD scores of 0.19, 0.26, and 0.28, respectively. **(C)** shows a 41-year-old female patient with HER2-zero breast cancer, with ITHT1, ITHT2, and ITHPD scores of 0.68, 0.67, and 0.24, respectively. The two-dimensional ROI is manually delineated on the synthetic T2-weighted images. ROI denotes the region of interest. ROI, region of interest.

The synthetic T1-weighted, T2-weighted, and PD-weighted images were imported into the open-source software ITK-SNAP (version 3.8.0, http://www.itksnap.org). Guided by DWI and DCE images, ROIs were manually delineated layer by layer along the medial side of the tumor margin on synthetic T2-weighted images containing the tumor. Subsequently, a multiscale radiomic technique developed by Li et al. for evaluating ITH in patients with non-small cell lung cancer was adapted for assessing ITH in breast cancer patients using MRI images (https://pypi.org/project/ITHscore) (16). The procedure for determining the ITH score comprised the following steps: (1) expanding the tumor area by 3 mm around the entire perimeter to include the peritumoral microenvironment; (2) extracting local radiomic features (including 19 first-order and 75 texture features) using a 3×3mm sliding window with the PyRadiomics software (version 3.1); (3) employing k-means clustering to identify subregions with similar radiomic feature distributions, with the elbow method employed to determine the optimal number of clusters for each tumor; and (4) calculating the ITH score (ITH_T1_, ITH_T2_ and ITH_PD_) by considering the number of connected regions and the largest area of these regions for each cluster. The ITH score ranges from 0 to 1. The final heterogeneous subregions and the corresponding ITH scores are illustrated in **Figure 1**.

### 2.6 Model development

In this study, machine learning models were constructed using variables identified as statistically significant through multivariate analysis. The research methodology comprised the following steps: (1) Patients were randomly assigned to training and validation cohorts in a 7:3 ratio; (2) Nine machine learning models were developed utilizing the statistically significant factors identified in the training dataset. These models included Random Forest (RF), Logistic Regression (LR), Extreme Gradient Boosting (XGBoost), Decision Tree (DT), Light Gradient Boosting Machine (LightGBM), Adaptive Boosting (AdaBoost), Gaussian Naive Bayes (GNB), Multilayer Perceptron (MLP), and Gradient Boosted Decision Trees (GBDT); (3) Optimal parameters for these models were determined using a retrospective 5-fold cross-validation approach. Model performance was assessed using core evaluation metrics including AUC, accuracy, sensitivity, and specificity. A predictive model was ultimately developed by integrating clinical data, MRI data, and the ITH score. Feature selection, including univariate screening and multivariate logistic regression, was performed exclusively on the training cohort and was completed prior to the 5-fold cross-validation procedure. The validation cohort was not used at any stage of feature selection or model training.

### 2.7 Statistical analysis

Statistical analyses were conducted using Python version 3.7. Categorical variables are presented as frequencies and percentages and were analyzed using the Chi-squared or Fisher’s exact tests. Continuous variables are reported as means and standard deviations and were examined using either the Mann–Whitney U test or Student’s t-test. Variables demonstrating statistically significant differences (P < 0.05) were subsequently incorporated into a multivariate logistic regression analysis. Clinical data, MRI data, and ITH scores that exhibited statistically significant differences (P < 0.05) were identified through multivariate analysis and employed to construct a predictive model. To evaluate the model’s performance, key metrics such as AUC, accuracy, sensitivity, specificity, positive predictive value (PPV), and negative predictive value (NPV) were utilized. To assess the practical applicability of the predictive model, Decision Curve Analysis (DCA) and Kolmogorov-Smirnov (KS) statistical plots were employed. The interpretation of the model was aided by SHAP analysis, which visualized how features influenced the model’s predictions.

## 3 Results

### 3.1 Patient characteristics

In this retrospective study, a cohort of 233 patients diagnosed with invasive breast cancer was analyzed. Among these patients, 78 (33.5%) exhibited HER2-zero expression, 72 (30.9%) demonstrated HER2-low expression, and 83 (35.6%) presented with HER2-positive expression. The three groups did not exhibit significant difference in menopausal status across all groups. Additionally, no statistically significant differences were observed in the maximum axial diameters of tumors among the groups, with median diameters of 26 mm, 21 mm, and 21 mm for patients with HER2-positive, HER2-low, and HER2-zero expression, respectively. As to status of ER and PR, our data demonstrate a statistically significant negative correlation between HER2 expression and both ER and PR expression. The detailed characteristics of the patients are presented in **Table 1**.

**Table 1:** Baseline characteristics.

Characters	HER2-negative	HER2-low	HER2-positive	P
Menopausal status				0.173
Premenopausal	60 (76.92)	48 (66.67)	53 (63.86)	
Postmenopausal	18 (23.08)	24 (33.33)	30 (36.15)	
Side				0.757
Left	55 (70.51)	49 (68.06)	61 (73.49)	
Right	23 (29.49)	23 (31.94)	22 (26.51)	
Breast composition				0.006
a	6 (7.69)	8 (11.11)	2 (2.41)	
b	32 (41.03)	16 (22.22)	16 (19.28)	
c	37 (47.44)	40 (55.56)	56 (67.47)	
d	3 (3.85)	8 (11.11)	9 (10.84)	
BPE				0.430
Minimal	62 (79.49)	46 (63.89)	54 (65.06)	
Mild	9 (11.54)	16 (22.22)	16 (19.28)	
Moderate	5 (6.41)	8 (11.11)	10 (12.05)	
Marked	2 (2.56)	2 (2.78)	3 (3.61)	
Non-mass enhancement				0.017
Present	77 (98.72)	66 (91.67)	72 (86.75)	
Absent	1 (1.28)	6 (8.33)	11 (13.25)	
Margin				< 0.001
Non-spiculated	36 (46.15)	28 (38.89)	11 (13.25)	
Spiculated	42 (53.85)	44 (61.11)	72 (86.75)	
Necrosis				0.768
Absent	66 (84.62)	62 (86.11)	68 (81.93)	
Present	12 (15.39)	10 (13.89)	15 (18.07)	
Rim enhancement				0.991
Absent	57 (73.08)	52 (72.22)	60 (72.29)	
Present	21 (26.92)	20 (27.78)	23 (27.71)	
Peritumoral edema				0.024
Absent	72 (92.31)	55 (76.39)	71 (85.54)	
Present	6 (7.69)	17 (23.61)	12 (14.46)	
Initial phase				
Slow or Medium	4 (5.13)	1 (1.39)	0 (0.00)	< 0.001
Rapid	74 (94.87)	71 (98.61)	83 (100.00)	
TIC				
I	3 (3.85)	8 (11.11)	1 (1.21)	0.043
II	41 (52.56)	28 (38.89)	39 (46.99)	
III	34 (43.59)	36 (50.00)	43 (51.81)	
ER				0.006
Negative	27 (34.61)	20 (27.78)	43 (51.81)	
Positive	51 (65.38)	52 (72.22)	40 (48.19)	
PR status				0.020
Negative	29 (37.18)	23 (31.94)	44 (53.01)	
Positive	49 (62.82)	49 (68.06)	39 (46.99)	
Age	52.13 (9.31)	53.54 (10.40)	50.53 (10.94)	0.194
Largest tumor size (mm)	21.00 (16.00, 28.00)	21.00 (15.00, 27.00)	26.00 (18.00, 37.00)	0.008
PD	80.71 (75.32, 86.59)	79.07 (73.87, 84.40)	77.46 (73.22, 80.39)	0.050
ITH_PD_	0.55 (0.18)	0.442 (0.21)	0.50 (0.21)	0.005
ITH_T2_	0.51 (0.42, 0.67)	0.43 (0.32, 0.53)	0.50 (0.33, 0.75)	0.009
ITH_T1_	0.51 (0.37, 0.67)	0.41(0.32, 0.64)	0.59 (0.48, 0.77)	0.002
ADC	0.91 (0.79, 0.99)	0.92 (0.80, 1.02)	1.02 (0.92, 1.10)	< 0.001
T1	964.22 (809.05, 1141.67)	984.00(893.00, 1138.00)	1024.82(827.01, 1152.34)	0.615
T2	72.19 (63.84, 82.00)	71.76 (65.39, 81.83)	70.80 (64.49, 75.25)	0.283

BPE, Background Parenchymal Enhancement; TIC, Time-Intensity Curve; ER, estrogen receptor; PR, progesterone receptor; PD, Proton Density; ITH, intratumoral heterogeneity; ITH_PD_, ITH_T2_, ITH_T1_: Intratumoral Heterogeneity (ITH) scores calculated using Proton Density (PD), T2 relaxation time, and T1 relaxation time, respectively; T1, T1 relaxation time; T2, T2 relaxation time.

**Figure 2:**
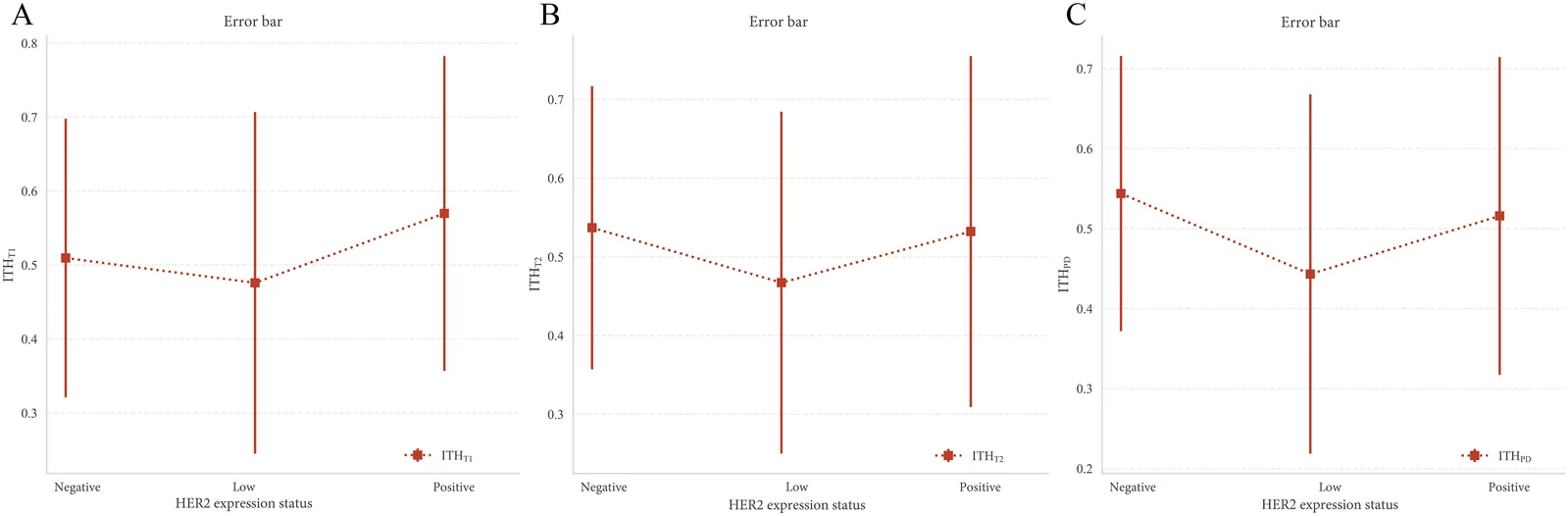
The relationship between ITH_T1_ **(A)**, ITH_T2_ **(B)**, and ITHPD **(C)** scores and HER2 expression. Intratumoral heterogeneity (ITH) scores were calculated using Proton density (PD), T2 relaxation time, and T1 relaxation time, respectively. A significant nonlinear correlation was observed between HER2 expression levels and ITH. The mean ITHT1 scores were 0.505, 0.414 and 0.591 in patients with HER2-negative, HER2-low and HER2-positive (P = 0.002). The mean ITH_T2_ scores were 0.514, 0.430 and 0.497 in patients with HER2-negative, HER2-low and HER2-positive (P = 0.009). The mean ITH_PD_ scores were 0.551, 0.442 and 0.498 in patients with HER2-negative, HER2-low and HER2-positive (P = 0.005).

Regarding the ITH score, a significant nonlinear correlation was observed between HER2 expression levels and ITH. The mean values of ITH_PD_, ITH_T2_, and ITH_T1_ were significantly lower in patients with HER2-low expression compared to those with HER2-positive and HER2-negative status (**Figure 2**).

### 3.2 Feature selection for model construction in the training data set

Univariate and multivariate analyses were employed to select features for model construction within the training dataset. In Task 1, four features were identified as statistically significant: the ITH_T1_ score (OR = 6.478, 95% CI: 1.043-43.997, P = 0.049), ADC value (OR = 37.431, 95% CI: 5.883-351.355, P < 0.001), age (OR = 0.964, 95% CI: 0.928-0.999, P = 0.049), and margin (OR = 3.490, 95% CI: 1.504-8.754, P = 0.005). In Task 2, four features were selected: the ITH_T1_ score (OR = 0.063, 95% CI: 0.009-0.411, P = 0.005), ADC value (OR = 0.032, 95% CI: 0.002-0.304, P = 0.005), ITH_PD_ score (OR = 0.133, 95% CI: 0.017-0.927, P = 0.046), and peritumoral edema (OR = 3.071, 95% CI: 1.055-9.328, P = 0.042). For Task 3, five features were identified: ITH_PD_ score (OR = 0.086, 95% CI: 0.016-0.429, P = 0.003), ADC value (OR = 11.210, 95% CI: 2.254-71.726, P = 0.006), initial phase (OR = 24.600, 95% CI: 2.154-708.990, P = 0.022), margin (OR = 2.419, 95% CI:1.252-4.728, P = 0.009), and peritumoral edema (OR = 2.989, 95% CI: 1.094-9.435, P = 0.044).

### 3.3 Construction and validation of model to distinguish HER2-positive versus HER2-negative tumors

**Figure 3:**
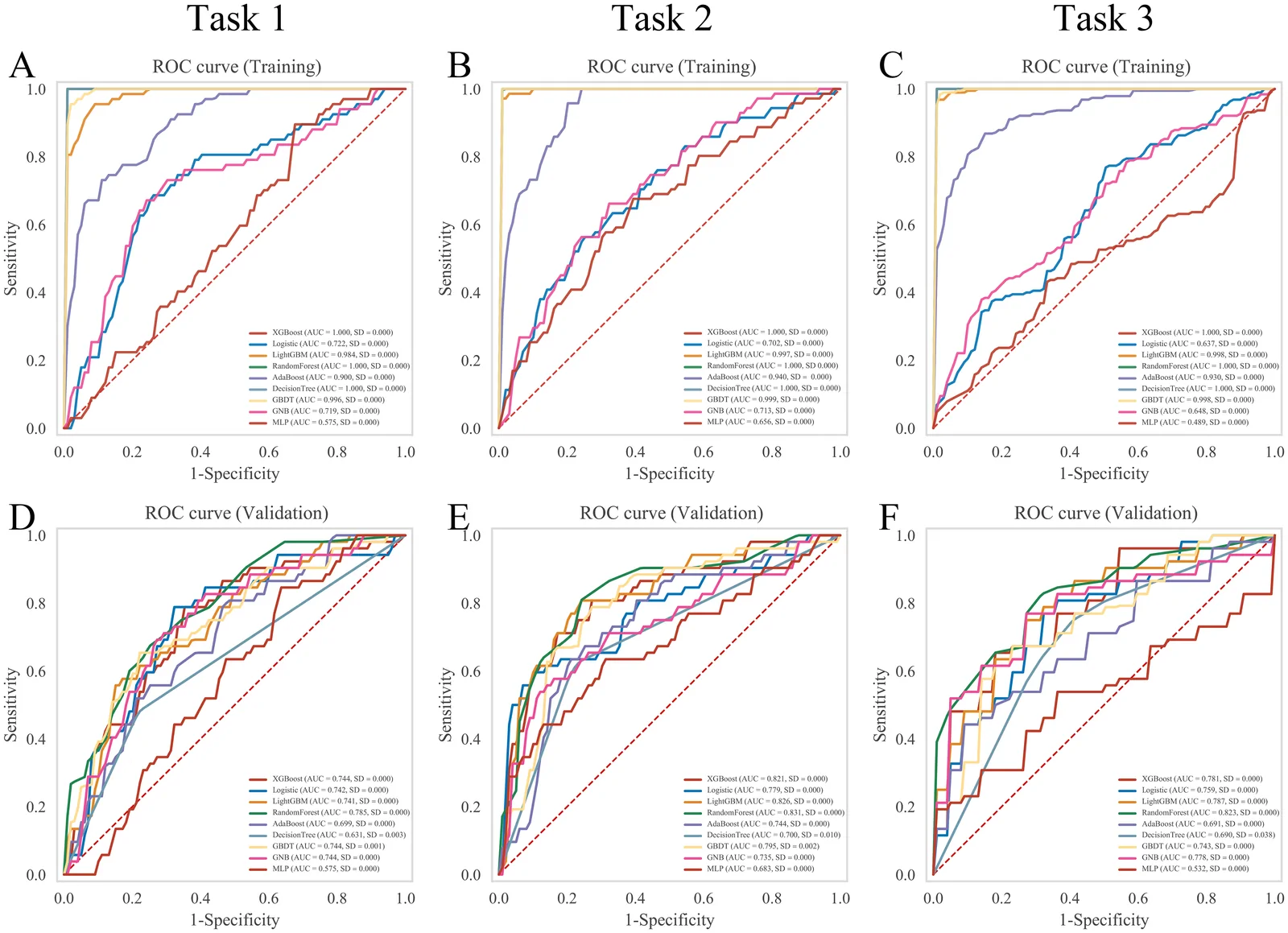
ROC curves of the nine machine learning models in training and validation sets. **(A, D)** were analysis for Task 1 aimed to differentiate HER2-positive and HER2-negative breast cancer. **(B, E)** were analysis for Task 2 focused on distinguishing HER2-low from HER2-zero/positive breast cancer. **(C, F)** were analysis for Task 3 involved differentiating between HER2-zero and HER2-low/positive breast cancer. ROC, Receiver operating characteristics; AUC, Area Under the Curve; XGB, EXtreme Gradient Boosting Classifier; LGBM, Light Gradient Boosting Machine Classifier; RF, RandomForest Classifier; GNB, Gaussian Naive Bayes; LR, Logistic Regression; MLP, Multilayer Perceptron Classifier; GBDT, Gradient Boosting Decision Tree.

**Figure 4:**
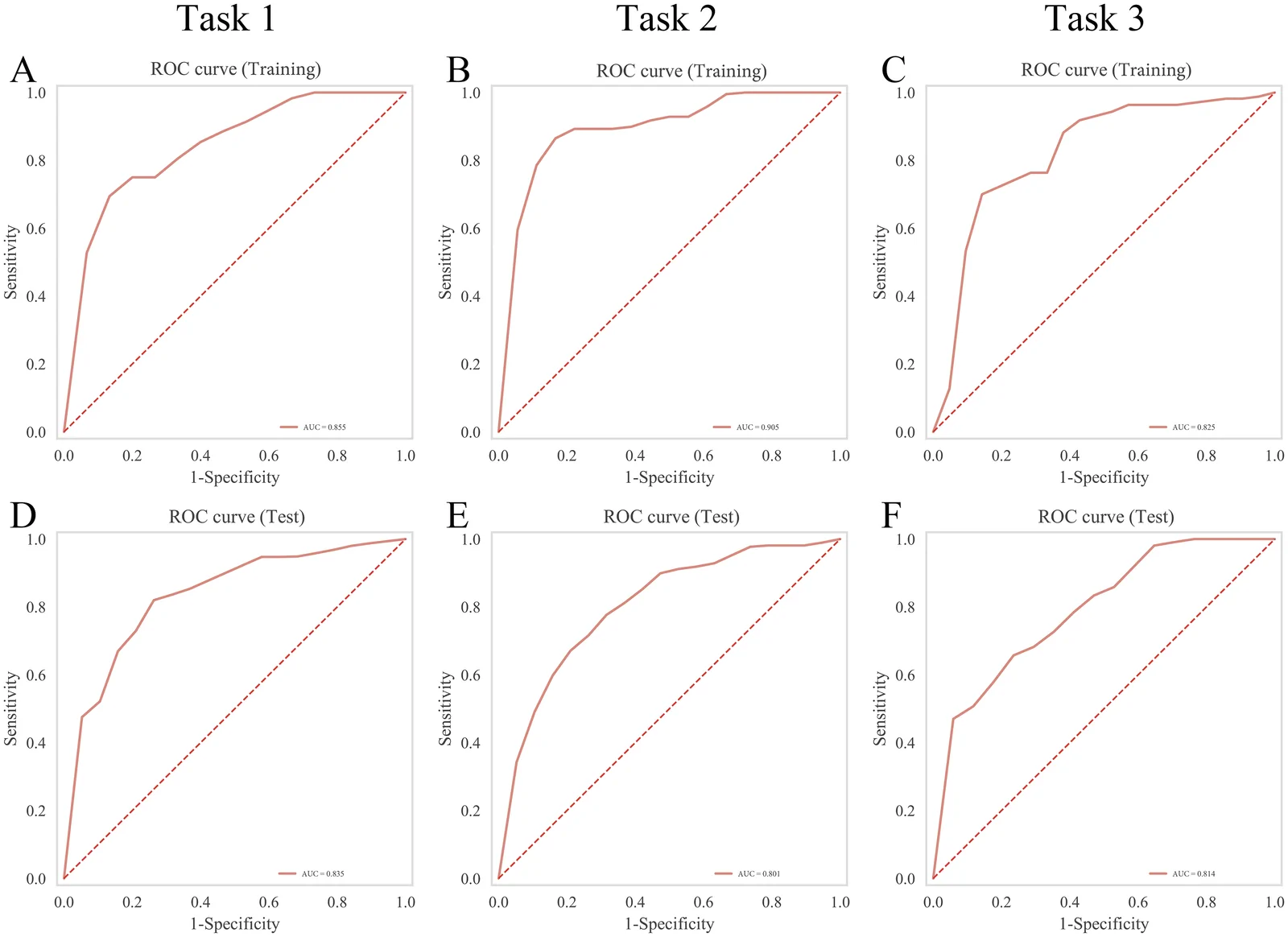
ROC curves of the RandomForest model for identifying HER2 subtypes in training and validation sets. **(A, D)** were analysis for Task 1 aimed to differentiate HER2-positive from HER2-negative breast cancer. **(B, E)** were analysis for Task 2 focused on distinguishing HER2-low from HER2-zero/positive breast cancer. **(C, F)** were analysis for Task 3 involved differentiating between HER2-zero and HER2-low/positive breast cancer. ROC, Receiver operating characteristics.

**Figure 5:**
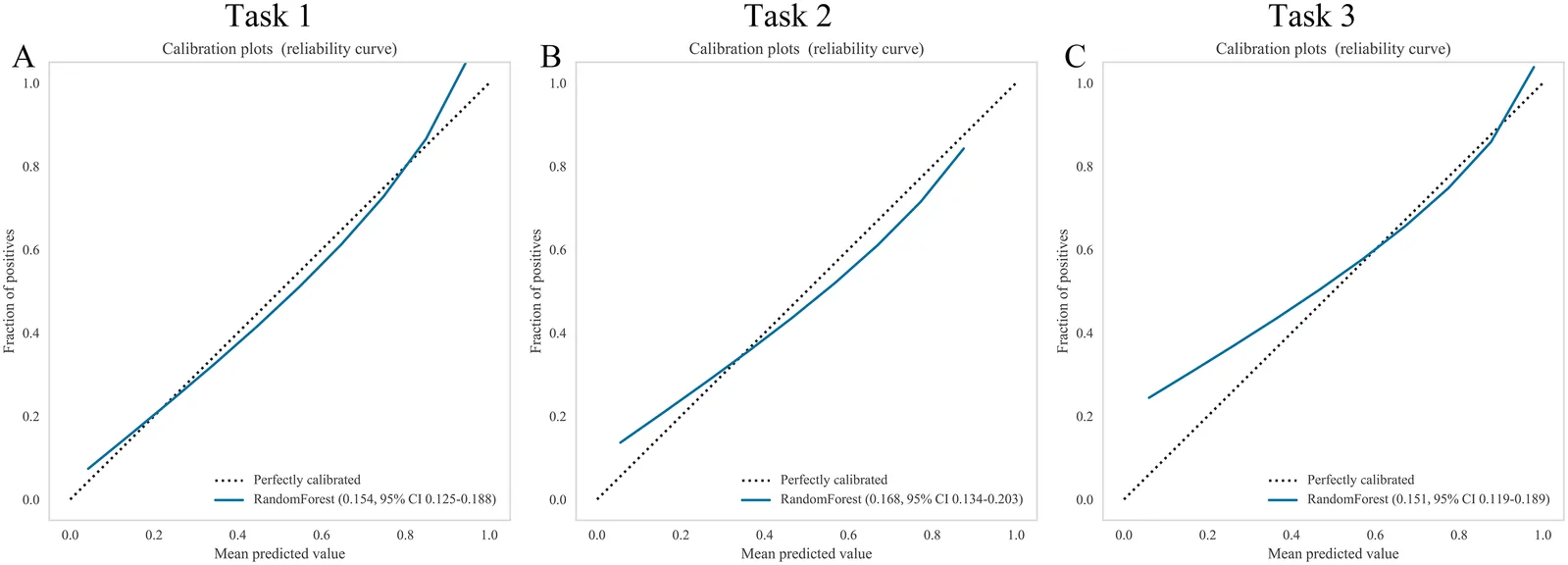
Calibration plot of the RandomForest model. **(A–C)** represent the calibration plot analyses for Task 1, Task 2, and Task 3, respectively.

**Figure 6:**
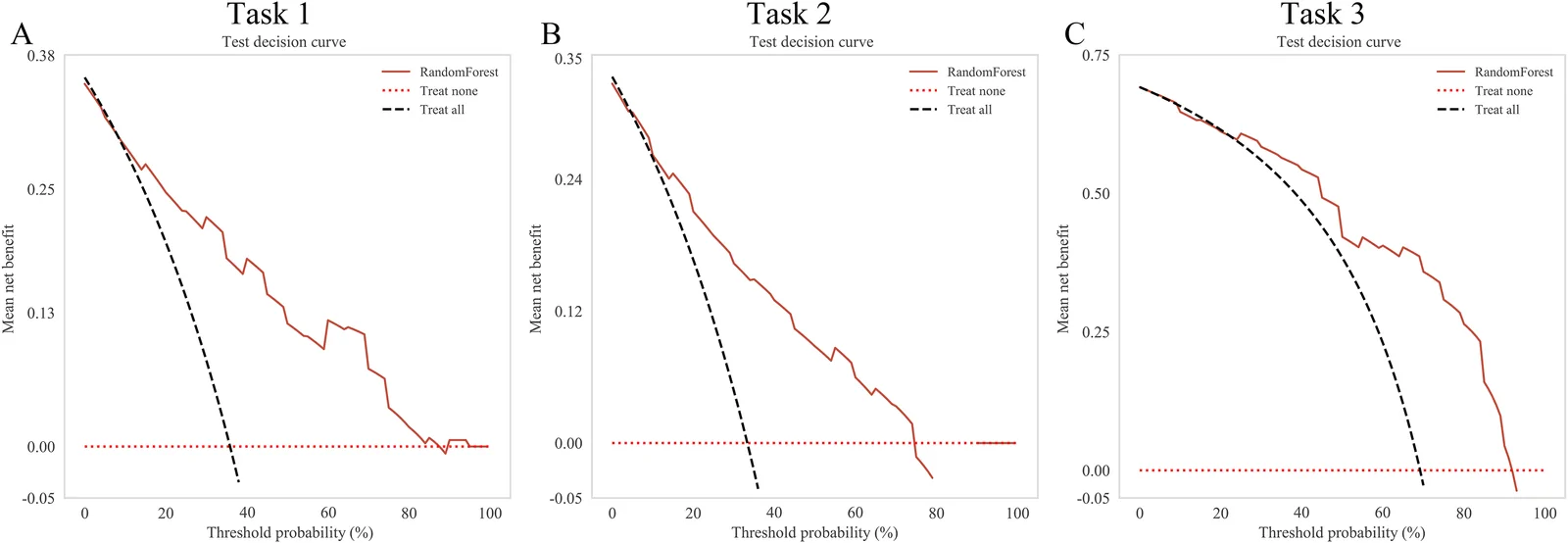
Decision curve analyses (DCA) of the RandomForest model. **(A–C)** represent the DCA analyses for Task 1, Task 2, and Task 3, respectively.

**Figure 7:**
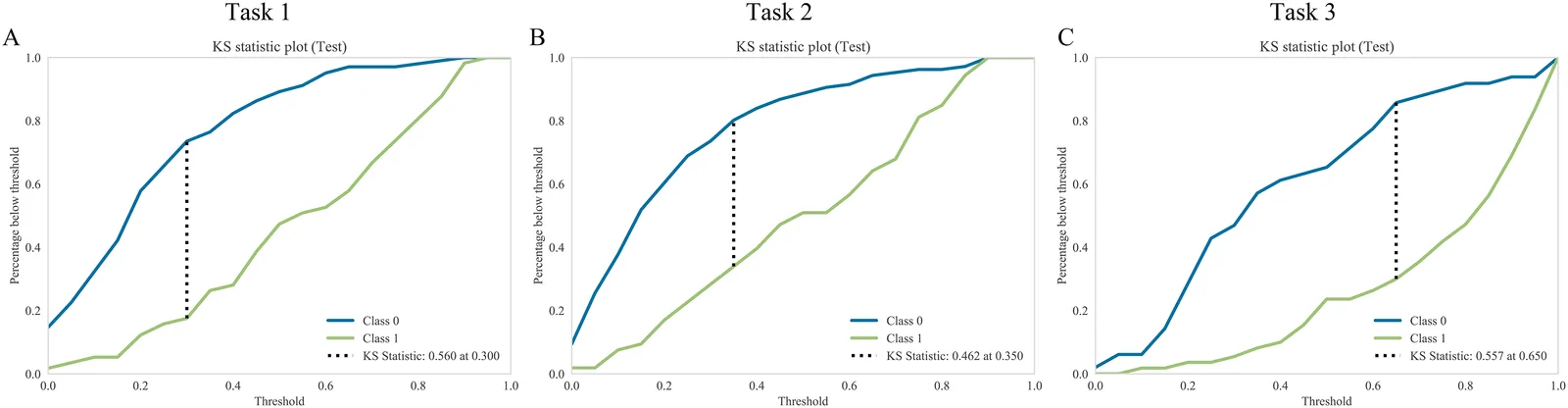
Kolmogorov-Smirnov (KS) statistical plots of the RandomForest model. **(A–C)** represent the KS analyses for Task 1, Task 2, and Task 3, respectively.

**Figure 8:**
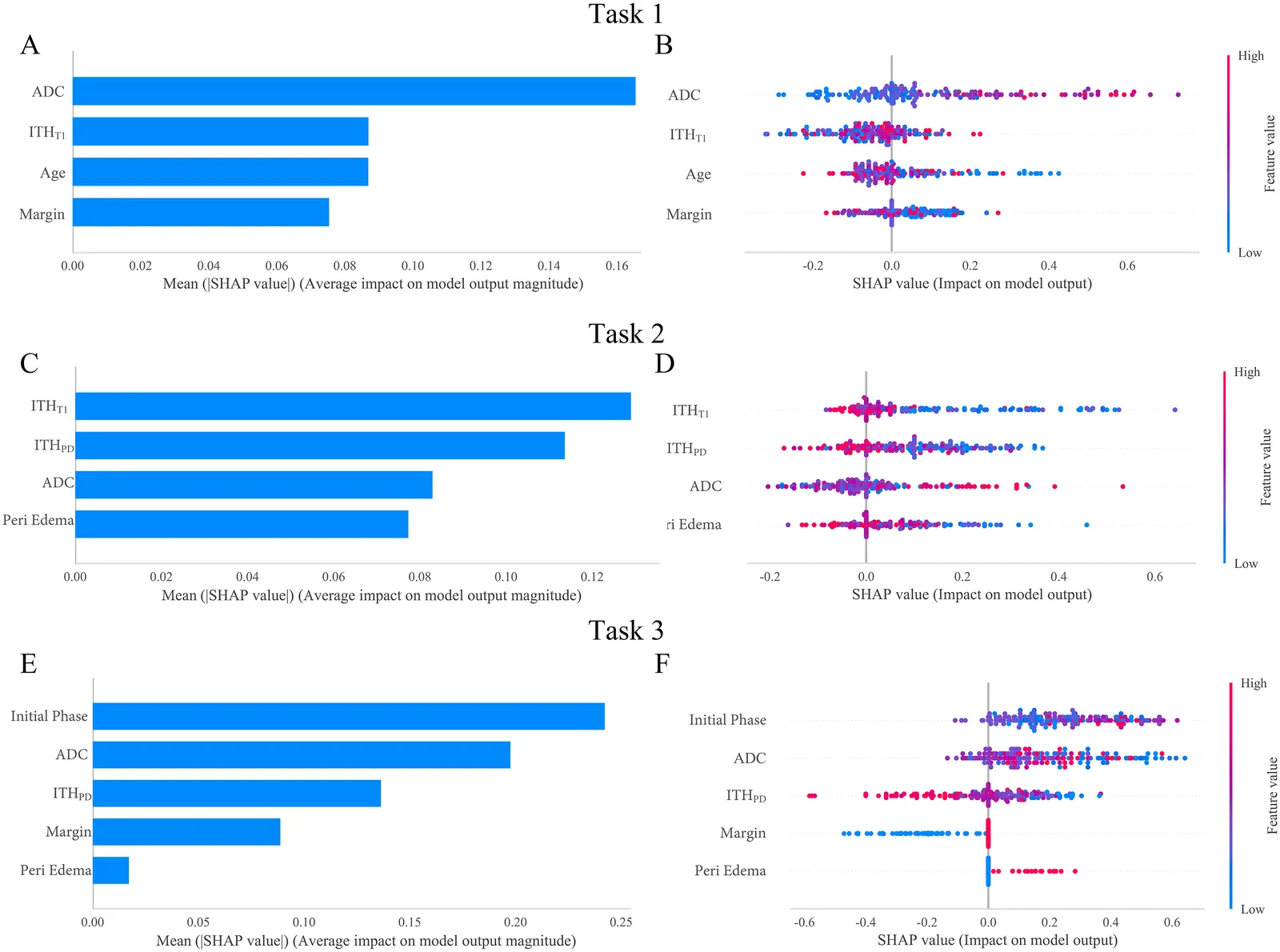
SHAP-based feature interpretation of three predictive models. **(A, C, E)** display bar plots of the mean absolute SHAP values, which rank the overall predictive significance of each feature for Tasks 1, 2, and 3, respectively. The horizontal axis represents the mean absolute SHAP value, indicating the average magnitude of each feature’s contribution to the model predictions. **(B, D, F)** illustrate SHAP summary dot plots for Tasks 1, 2, and 3, respectively, where each dot corresponds to an individual sample. The horizontal axis indicates the SHAP value, which measures the positive or negative impact of a feature on the model output. The color gradient from blue to red represents the raw values of the corresponding feature, ranging from low to high. SHAP refers to Shapley Additive Explanations analysis. Peri Edema, peripheral edema.

In the training dataset, nine machine learning prediction models were developed based on the selected features, including RF, LR, XGBoost, DT, LightGBM, AdaBoost, GNB, MLP, and GBDT. Among these models, the RF model exhibited superior performance, achieving an AUC value of 1.000 for the training group and 0.785 for the validation group (**Figures 3A, D**), with greater stability on the validation set (lower variance across 5-fold cross-validation) and better prevention of overfitting compared to other models. The RF model, which incorporated four specific parameters identified in Task 1, attained AUC values of 0.855 for the training group and 0.835 for the validation group (**Figures 4A, D**), with a Brier score of 0.154, as shown in **Figure 5A**. The DCA revealed that the net benefit of the combined model for the prediction of HER2-positive was high when the probability of the threshold was set over 20% (**Figure 6A**). The KS statistical chart validated the model’s efficacy, yielding a KS value of 0.560 at the optimal prediction probability threshold (**Figure 7A**). The SHAP bar chart provided a comprehensive overview of the four features by calculating the mean absolute SHAP values for each. The SHAP beeswarm plots illustrated the positive and negative influences of each feature on the prediction probability, employing red and blue colors to represent these effects across both tasks. Among the features, the ADC value exerted a more significant impact on differentiating between HER2-positive and HER2-negative statuses compared to the other features (**Figures 8A, B**).

### 3.4 Construction and validation of model to distinguish HER2-low versus HER2-positive/zero tumors

In the training dataset, among the nine machine learning prediction models, the RF model exhibited superior performance, achieving AUC values of 1.000 for the training group and 0.831 for the validation group (**Figures 3B, E**). The RF model, which incorporated four specific parameters, attained AUC values of 0.905 for the training group and 0.801 for the validation group (**Figure 4B** and 4E), with a Brier score of 0.168, as shown in **Figure 5B**. The DCA revealed that when the probability of the threshold was set over 20%, the net benefit of the combined model for the prediction of HER2-Low was high (**Figure 6B**). The KS statistical chart validated the model’s efficacy, yielding a KS value of 0.462 at the optimal prediction probability threshold (**Figure 7B**). The SHAP analysis revealed that both the ITHT1 and ITHPD scores had a significant impact on the predicted probabilities for differentiating between HER2-zero and HER2-positive/low status (**Figures 8C, D**).

### 3.5 Construction and validation of model to distinguish HER2-zero versus HER2-positive/low tumors

In the training dataset, among the nine machine learning prediction models, the RF model exhibited superior performance, achieving AUC values of 1.000 for the training group and 0.823 for the validation group, as detailed in **Figures 3C, F**. The RF model, which incorporated five specific parameters, attained AUC values of 0.825 for the training group and 0.814 for the validation group (**Figures 4C, F**), with a Brier score of 0.151, as shown in **Figure 5C**. The DCA revealed that when the probability of the threshold was set over 30%, the net benefit of the combined model for the prediction of HER2-negative was high (**Figure 6C**). The KS statistical chart validated the model’s efficacy, yielding a KS value of 0.557 at the optimal prediction probability threshold (**Figure 7C**). The SHAP analysis indicated that the initial phase significantly affected these predicted probabilities for distinguishing between HER2-zero and HER2-positive/low status (**Figures 8E, F**).

## 4 Discussion

As the classification of traditional HER2 status continues to evolve, there is a growing demand for precise non-invasive methodologies to distinguish among HER2 subtypes in breast cancer. In this retrospective study, we investigated the utility of ITH scores derived from SyMRI for non-invasive differentiation of HER2 subtypes in breast cancer. By integrating these quantitative ITH scores with MRI image features and clinical characteristics, we developed three models that demonstrated robust performance in differentiating between HER2-positive and HER2-negative cases, HER2-low and HER2-positive/zero cases, as well as HER2-zero and HER2-positive/low groups.

The models were trained using data from 163 patients and subsequently validated with an independent cohort of 70 patients, ensuring no overlap with the training dataset. The features incorporated into the models for task 1, task 2, and task 3 were as follows: four features (ITH_T1_ score, ADC value, age, and margin) for task 1; four features (ITH_T1_ score, ADC value, ITH_PD_ score, and peritumoral edema) for task 2; and five features (ITH_PD_ score, ADC value, initial phase, margin, and peritumoral edema) for task 3. The findings also indicate a significant nonlinear correlation between ITH scores and HER2 expression levels. Notably, the mean values of ITH_PD_, ITH_T1_, and ITH_T2_ were significantly lower in patients with HER2-low compared to those with HER2-positive and HER2-negative status, a novel observation. This provides a basis for developing a model capable of distinguishing between HER2-low, HER2-positive, and HER2-negative tumors.

Recently, the SyMRI technique has been developed to simultaneously quantify T1 and T2 relaxation times, as well as PD images, through a comprehensive pulse sequence that utilizes multiecho and multidelay acquisition methods (17). The clinical application of SyMRI technology has extended beyond the central nervous system, with its use in distinguishing benign from malignant breast diseases and in molecular typing assessments becoming increasingly prominent (9, 18). In this study, to enhance the stability and predictive capability of the models, the calculation of the ITH score employed an unsupervised clustering algorithm that does not depend on large sample sizes. The entire tumor region was segmented into three habitat subregions with similar features using K-means clustering. We extracted T1, T2, and PD values from enhanced SyMRI sequences and found that these values did not exhibit statistically significant differences among the three groups, consistent with the findings of Du et al. (19). The ITH scores derived from T1, T2, PD, and ADC were significantly associated with HER2 status and may enhance the robustness of predictive models, as these features are quantitative and more objective compared to traditional MRI features.

The SHAP analysis further elucidated the differential contribution of individual features across the three prediction tasks. For Task 1 (distinguishing HER2-positive from HER2-negative), the ITH_T1_ score and ADC value emerged as the most influential predictors. The importance of ADC likely reflects the enhanced angiogenesis characteristic of HER2-amplified tumors, which increases tissue perfusion and elevates the apparent diffusion coefficient (20). In Task 2 (differentiating HER2-low from HER2-zero/positive), ITH_T1_ and ITH_PD_ were the dominant features, suggesting that the synthetic T1-weighted and PD-weighted heterogeneities captures subtle differences in tumor composition that distinguish HER2-low tumors from other subtypes. For Task 3 (separating HER2-zero from HER2-low/positive), the initial phase showed the greatest impact, indicating that the early enhancement pattern on dynamic MRI provides discriminative information for identifying HER2-zero tumors, which tend to exhibit less prominent early vascular enhancement. Together, these SHAP findings demonstrate that the model does not rely on a single feature type but integrates complementary information from both tissue heterogeneity (ITH scores) and physiological properties (ADC, initial phase), each contributing differentially across the three classification tasks.

Previous studies have demonstrated that ITH features emerge from the heterogeneous composition of cell populations and their uneven distribution within a tumor (21, 22). Imaging techniques facilitate the direct quantification of tumor phenotypes at various spatial resolutions (23). Notably, the habitat subregions identified through K-means clustering may correspond to biologically distinct tumor microenvironments. As noted by Gillies et al., imaging-defined tumor subregions, or “habitats,” can reflect the heterogeneous perfusion and cellular composition within a tumor (13). Similarly, Shi et al. demonstrated that MRI-based quantification of intratumoral subregion diversity was independently associated with treatment response in breast cancer (15). In the present study, the clustering-derived ITH scores provide a quantitative measure of intratumoral spatial heterogeneity, potentially capturing differences in tumor architecture that are characteristic of different HER2 subtypes. Direct histopathological validation of each habitat subregion warrants further investigation.

As an emerging biomarker, the ITH score offers a more comprehensive representation of ITH than traditional radiomics and is widely applied in diverse areas of cancer research. Additionally, prior research on habitat radiomics has elucidated the associations between SyMRI and HER2 expression. In our current study, ADC values showed a gradual increase in breast cancer patients with HER2-negative, weakly positive, and positive expression. A previous study demonstrated the significance of the ADC value in diagnosing HER2-positive/hormone receptor-negative (HR−) breast cancer, reporting an AUC of 0.839 (95% CI: 0.791-0.887) (P < 0.01) (24). This phenomenon could be explained by that the angiogenic ability of HER2+ breast cancer is stronger, and the tissue perfusion ability is faster than the increase of tumor cell density, and its ADC value is thus higher (20).

Traditional MRI features such as initial phase, margin, and peritumoral edema have been extensively studied, and previous research has indicated that DCE-MRI characteristics are frequently employed to describe tumor morphology and enhancement patterns in breast cancer evaluation. For instance, one study revealed that HER2+ breast cancers within the HR− subtype more often presented as non-mass enhancement (NME) or as a mass with NME, whereas the HR+ subtype typically manifested as a mass. Additionally, HR− cases were more likely to display round or oval masses with circumscribed margins, while HR+ cases commonly exhibited irregular or spiculated margins (24). Nevertheless, the existing literature does not explicitly differentiate the initial phase, such as specific stages of early enhancement rate. It merely notes that the early enhancement rate and time-signal intensity curves (TIC) exhibit no significant differences among HER2+ subtypes (24). As for peritumoral edema, it is not directly addressed in the literature. However, one study does indicate that necrotic areas appear as regions of high signal intensity on T2-weighted sequences, which may indirectly pertain to edema. Despite this, no focused analysis has been conducted specifically on peritumoral edema (25).

Currently, research efforts aimed at predicting HER2 expression status have predominantly concentrated on MRI-based approaches. Guo et al. developed a deep learning radiomic model utilizing MRI to specifically distinguish between HER2-low and HER2-zero expression, achieving an AUC of 0.750 (26). Nonetheless, the deep learning radiomic model is limited to identifying HER2-negative status. In a related study, Ramtohul et al. developed an MRI-based radiomic model to differentiate HER2-zero expression from other expressions (HER2-low expression and HER2-over expression), achieving an AUC of 0.80 (27). However, this study did not develop models to distinguish HER2-over expression from other expressions or to differentiate HER2-low expression from other categories. In this study, we integrated clinical independent predictors with ITH scores derived from SyMRI to conduct three comparative analyses: HER2-positive versus HER2-negative, HER2-low versus HER2-zero/positive, and HER2-zero expression versus HER2-low/positive. The robustness of our model is demonstrated by its ability to accurately predict HER2-positive versus HER2-negative status (AUCs of 0.855 and 0.835) and to effectively differentiate HER2-low from HER2-zero/positive (AUCs of 0.905 and 0.801), as well as HER2-zero from HER2-low/positive (AUCs of 0.825 and 0.814). Our findings are comparable to existing studies, and our predictive models are relatively simple yet competitive when compared to previously reported models. Although a formal head-to-head comparison was beyond the scope of this study, several conceptual differences merit discussion. Conventional radiomics extracts features from the entire tumor volume, potentially averaging out subregional heterogeneity. In contrast, the habitat-based ITH approach partitions the tumor into phenotypically distinct subregions before feature extraction, preserving the spatial organization relevant to HER2 subtype classification. However, the additional clustering step introduces complexity that may reduce reproducibility across centers compared to whole-tumor radiomics. Future studies directly comparing the two pipelines would help quantify the incremental value of habitat decomposition.

The present study is subject to several limitations that warrant consideration. First, this was a small-scale retrospective single-center study, and no suitable publicly available dataset containing synthetic MRI quantitative maps (T1, T2, and PD) was available for external validation. Therefore, the generalizability of our findings should be further confirmed in independent multicenter cohorts. Second, although unsupervised clustering identified the habitat subregions, direct histopathological or molecular validation of each subregion was not feasible due to retrospective constraints and technical complexities of multi-regional sampling. Therefore, the precise biological significance of these imaging habitats requires further translation. Third, our exclusive reliance on synthetic MRI sequences limited the comprehensive understanding of tumor biology. To enhance the model’s accuracy, future studies will incorporate multi-parametric imaging modalities, such as DCE-MRI and DWI. Fourth, the manual segmentation employed to delineate the ROI in this study was both labor-intensive and time-consuming, potentially introducing errors. Therefore, improving efficiency and precision necessitates the development of advanced tumor segmentation techniques or automated identification tools. To enhance the reliability of future research, stricter sample screening, increased sample sizes, addition of radiomics features, study on the relationship between the cluster features and biology and expanded multicenter collaborations will be implemented.

## 5 Conclusions

In summary, the habitat model developed from SyMRI sequence images in this study is capable of quantifying ITH for the evaluation of HER2 status. Furthermore, by incorporating clinical independent predictors with the ITH score, our model demonstrates a high level of accuracy in predicting the HER2 status of breast cancer, thereby providing substantial support for clinical decision-making.

## Data Availability

The raw data supporting the conclusions of this article will be made available by the authors, without undue reservation.
